# Intravenous *Mycobacterium Bovis* Bacillus Calmette-Guérin Ameliorates Nonalcoholic Fatty Liver Disease in Obese, Diabetic *ob/ob* Mice

**DOI:** 10.1371/journal.pone.0128676

**Published:** 2015-06-03

**Authors:** Masashi Inafuku, Goro Matsuzaki, Hirosuke Oku

**Affiliations:** 1 Department of Tropical Bio-resources, Tropical Biosphere Research Center, University of the Ryukyus, Nishihara, Okinawa, Japan; 2 Department of Infectious Diseases, Tropical Biosphere Research Center, University of the Ryukyus, Nishihara, Okinawa, Japan; Nihon University School of Medicine, JAPAN

## Abstract

Inflammation and immune response profoundly influence metabolic syndrome and fatty acid metabolism. To analyze influence of systemic inflammatory response to metabolic syndrome, we inoculated an attenuated vaccine strain of *Mycobacterium bovis* Bacillus Calmette–Guérin (BCG) into leptin-deficient *ob/ob* mice. BCG administration significantly decreased epididymal white adipose tissue weight, serum insulin levels, and a homeostasis model assessment of insulin resistance. Serum high molecular weight (HMW) adiponectin level and HMW/total adiponectin ratio of the BCG treated mice were significantly higher than those of control mice. Hepatic triglyceride accumulation and macrovesicular steatosis were markedly alleviated, and the enzymatic activities and mRNA levels of lipogenic-related genes in liver were significantly decreased in the BCG injected mice. We also exposed human hepatocellular carcinoma HepG2 cells to high levels of palmitate, which enhanced endoplasmic reticulum stress-related gene expression and impaired insulin-stimulated Akt phosphorylation (Ser473). BCG treatment ameliorated both of these detrimental events. The present study therefore suggested that BCG administration suppressed development of nonalcoholic fatty liver disease, at least partly, by alleviating fatty acid-induced insulin resistance in the liver.

## Introduction

Obesity, especially visceral obesity, contributes to the pathogenesis of the metabolic syndrome, a cluster of metabolic abnormalities that includes hyperlipidemia, type 2 diabetes mellitus (T2DM), and hypertension [1]. The metabolic syndrome is a widespread and an increasingly prevalent disease in both developed and developing countries, and contributes to an increase in the rates of morbidity and mortality by cardiovascular disorders [1]. Nonalcoholic fatty liver disease (NAFLD) is also associated with metabolic syndrome. It encompasses a wide spectrum of disease from simple hepatic steatosis to steatohepatitis, advanced fibrosis, and cirrhosis [2]. It has recently been reported that various immune cells play key roles in the development of obesity-related metabolic abnormalities [3]. Obesity-related insulin resistance (IR) is also associated with elevated cytokine levels, including tumor necrosis factor (TNF)-*α* and interleukin (IL)-6, and elevated serum free fatty acid levels [4, 5].

Recent studies have demonstrated altered immune function in obese human compared with that in healthy patients compared with healthy individuals, suggesting that obesity may result in altered immune surveillance and in an impaired host defense [6]. Decreased resistance against viral,bacterial, and fungal infections has been shown in genetic- and diet-induced obesity model animals [7–9]. Many animal studies have shown that both type 1 diabetes mellitus (T1DM) and T2DM lead to increased susceptibility to infection with *Mycobacterium tuberculosis* (Mtb), which is responsible for human tuberculosis (TB) [10, 11].

One-third of the world’s population is infected with Mtb, with over 9 million new cases and 1.5 million deaths estimated from TB in 2013 [12]. An attenuated strain of *M*. *bovis* Bacillus Calmette–Guérin (BCG) is used worldwide as a vaccine against TB. Not only have cohort studies demonstrated that diabetes is a moderate-to-strong risk factor for the development of active TB [13], but the severity of diabetes has also been suggested to increase the risk of TB. Conversely, obesity is associated with a lower risk of active pulmonary TB, although that finding did not extend to extra-pulmonary TB [14]. Human T1DM is caused by an absolute deficiency of insulin production by pancreatic β–cells due to autoimmune T cell-induced destruction. This T-cell–dependent autoimmunity against islets β-cells plays an equally central role in the pathogenesis of nonobese diabetic (NOD) mouse models, which are used as an animal model for human T1DM.

It has been reported that BCG administration can prevent insulitis and T1DM in NOD mice [15, 16], and recent clinical trial data has suggested that BCG treatment can ameliorate human T1DM by stimulating the host innate immune response [17]. Interferon (IFN)-γ produced from mycobacterial antigen-specific T cells are considered important in the protective immunity against Mtb infection [18], being released as a first-line host defense mechanism after BCG vaccination [19]. TNF-*α* is another important cytokine in protective immunity against Mtb infection [20], and BCG administration is well known to induce TNF-*α* production [19]. TNF-*α* treatment prevents some autoimmune diseases including T1DM because autoreactive T cells were more susceptible to TNF-*α*-induced apoptosis than healthy T cells [21]. BCG administration may prevent T1DM by the TNF-*α*-induced apoptosis of diabetogenic T cells [17, 22]. In contrast, TNF-*α* also appears to be important in the progression of metabolic disorders such as IR and T2DM [4].

These results led us to propose that BCG treatment likely modulates a state of metabolic syndrome. However, no study has reported the effect of mycobacteria on the pathogenesis of obesity-related metabolic disorders. Although one study did report the effect of intranasal Mtb infection in leptin-deficient *ob/ob* mice, it only examined the immune response to TB [23]. Leptin is well known as a key mediator of energy metabolism; in addition, it is now recognized to play a role in the immune system. Wieland *et al*. suggested that leptin plays a role in early immune response to pulmonary TB [23]. Therefore, in this study, we aimed to examine the effects of intravenous (i.v.) BCG administration on the development of metabolic syndrome in leptin-deficient *ob/ob* mice.

## Materials and Methods

### Microorganisms and animals

BCG was purchased from Japan BCG (Tokyo, Japan), and suspended at 1×10^8^ colony-forming units (CFU)/mL in phosphate-buffered saline (PBS) before use.

All animal experimental protocols were approved by the University of the Ryukyus Animal Experiment Committee (Permit Number: 5488), and the experiments were conducted according to the ethical guidelines for animal experiments of the University of the Ryukyus. Twelve 5-wk-old male *ob/ob* mice were purchased from Japan SLC, Inc. (Shizuoka, Japan) and housed individually in plastic cages under specific pathogen-free conditions maintained at 24°C in a 12 h light–dark cycle. After one week of adaptation, the mice were randomly divided into a control group and a BCG group (6 per group). Next, a 20-fold human dose of of BCG (1 × 10^7^ CFU) was injected (i.v.) into *ob/ob* mice of BCG group. Control mice were injected with 100 μL of PBS. The mice were fed a commercial powdered chow (CE-2, CLEA Japan, Inc., Tokyo, Japan) and permitted water ad libitum for 4 weeks. At the end of the feeding period, the mice were killed after 12 h of starvation by exsanguination from the heart under anesthesia with pentobarbital sodium to minimize suffering. Their livers and white adipose tissues (WAT; epididymal, perirenal, omental, and waist subcutaneous) were excised, and serum was separated from the blood. A part of the excised livers was fixed in 10% neutral formalin solution, and the remaining liver and sera were frozen immediately in liquid nitrogen and then were stored at −80°C until use.

### Cell culture

Human hepatoma cell line, HepG2, were purchased from JCRB Cell Bank (Tokyo, Japan) and maintained in Dulbecco’s modified Eagle’s medium (DMEM) containing low glucose (1000 mg/L) supplemented with 10% fetal bovine serum. Then, 24 h after seeding into a 48-well plate (1 × 10^5^ cells per well), cells were co-cultured with 1 × 10^6^ CFU of BCG for 24 h. HepG2 cells were then treated in serum-free DMEM (low glucose) supplemented with 0.5 mM palmitate in the presence of 1 × 10^6^ CFU of BCG for 18 h. At the end of the treatment, cells were incubated with 100 nM insulin for 15 min.

### Histopathological examination of the livers

Formalin-fixed liver samples were embedded in paraffin and cut into 4-μm sections. The paraffinized tissue sections were stained with hematoxylin and eosin (HE) for microscopic evaluation of the degree of NAFLD. Steatosis score is determined by the sum of scores of macrosteatosis, microsteatosis, and hypertrophy according to the method described by Liang *et al*. [24]. The score (0 to 3) of macrosteatosis, microstetosis, and hypertrophy were determined on three randomly selected fields of each liver section, and the sum of these scores (steatosis score) was calculated for each fields. The mean of the scores fo these three fields was calculated as the steatosis score for the specimen, and the liver histology of 6 mice per group was examined.

### Measurement of biochemical parameters in serum and culture media

Triglyceride (TG), total cholesterol (TC), free fatty acids (FFA), and glucose levels were determined using a commercial enzymatic kit (Wako Pure Chemical Industries, Ltd., Osaka, Japan). Serum insulin and β-hydroxybutyrate levels were measured using enzyme-linked immunosorbent assay (ELISA) kits purchased from Morinaga Institute of Biological Science, Inc. (Kanagawa, Japan) and Abcam plc (Cambridge, UK), respectively. Total and high molecular weight (HMW) adiponectin levels were measured in serum by ELISA (APLICO Diagnostics, NH, USA). Serum cytokine levels of IL-6, monocyte chemoattractant protein-1 (MCP-1), TNF-α and IFN-γ were determined by cytometric bead assay (BD biosciences, CA, USA).

### Measurement of TG and TC levels in the liver

Hepatic lipids were extracted and purified according to a method reported previously [25]. We determined hepatic TG levels using the method described by Fletcher [26] and hepatic TC levels using commercial enzymatic kits (Wako Pure Chemical Industries).

### Assay of enzymatic activities in the livers

The preparation of hepatic subcellular fractions was performed according to methods described elsewhere [27]. The protein concentration of each fraction was determined using a Quant–iT protein assay kit (Life technologies, CA, USA). We measured the activities of fatty acid synthase (FAS), malic enzyme (ME), and glucose-6-phosphate dehydrogenase (G6PD) in cytosolic fraction, and that of carnitine palmitoyltransferase (CPT) in mitochondrial fraction, as described elsewhere [28].

### Western blotting

Protein was extracted from cultured cells using PRO-PREP (iNtRON Biotechnology, Inc., Gyeonggi-do, Korea) and stored at −80°C until use. Total protein concentration was determined using a Quant–iT protein assay kit. Equal amounts of protein were separated by sodium dodecylsulfate-polyacrylamide gel electrophoresis, and transferred onto polyvinylidene diflouride membrane. The blots were probed with antibodies against Akt (pan) and p-Akt (Ser 473) (Cell Signaling Technology, Tokyo, Japan) according to the manufacture’s protocol. The membrane was washed and incubated with horseradish peroxidase-conjugated secondary antibody (Biosource international, Inc., CA, USA) for 2 h at room temperature. The protein bands were developed with the ECL Prime Western Blotting Detection System (GE Healthcare, Buckinghamshire, England) and visualized using ImageQuant LAS4000 mini (GE Healthcare). The band intensities were quantified with ImageJ software (NIH, MA, USA).

### Quantitative real-time polymerase chain reaction and analysis of XBP1 splicing

Total RNA was extracted from the excised liver and cultured cells using a TRIzol reagent and a PureLink RNA mini kit (Life technologies). First strand cDNA was synthesized with 2 μg of total RNA as a template. In quantitative real-time polymerase chain reaction (PCR), we used the following murine genes: 18S rRNA (*Rn18s*), acetyl-coenzyme A (CoA) carboxylase α and β (*Acaca* and *Acacb*), fatty acid synthase (*Fasn*), steroyl CoA desaturase 1 (*Scd1*), malic enzyme 1 (*Me1*), glucose-6-phospate dehydrogenase (*G6pdx*), carnitine palmitoyltransferase 1a (*Cpt1a*), hormone sensitive lipase (*Lipe*), acyl- CoA oxidase (*Acox*), uncoupling proteins 2 (*Ucp2*), and adiponectin receptor 1 and 2 (*Adipor1* and *Adipor2*). We used the following human genes: β-actin (*ACTB*), fatty acid synthase (*FASN*), steroyl-CoA desaturase 1 (*SCD1*), acetyl-CoA carboxylase alpha (*ACACA*), acetyl-CoA carboxylase beta (*ACACB*), binding immunoglobulin protein (*BIP*), C/EBP homologous protein (*CHOP*), and X-box binding protein (*XBP1*). The amplifications were performed using the StepOne Real-Time PCR System (ABI). The relative amount of each gene transcript was normalized to the amount of *Rn18s* in mouse liver and to *ACTB* in the cultured human cells. To analyze the *XBP1* mRNA splicing, the PCR products were amplified using the specific primer, and digested with *PstI* before being separated in an agarose gel according to a method described elsewhere [29].

### Statistical analysis

Data are expressed as means ± standard error of the mean (SEM). The statistical significance of the difference between two experimental groups was determined using student *t*-tests. To determine the significance of the differences among means for more than three groups, the differences among the mean values were inspected using Tukey–Kramer’s multiple comparison test. Differences were considered significant at *P* < 0.05.

## Results

### Effect of BCG on growth parameters, lipid, β-hydroxybutyrate, and cytokines

We assessed the effect of BCG administration on growth parameters, and serum lipid, β-hydroxybutyrate, and cytokine levels in *ob/ob* mice. No significant differences were observed in food intake, initial and final body weights, or relative liver weights (Table 1). Although total, perirenal, omental and subcutaneous WAT had weights between the experimental groups, the relative weight of epididymal WAT in the BCG group was significantly lower than that in the control group. Serum lipid and β-hydroxybutyrate levels were largely comparable between the experimental groups (Table 2), but cytokine levels in serum were markedly increased in a BCG group compared with a control group (Table 2). The latter suggested successful BCG infection and induction of immune response against BCG.

**Table 1:** Effects of Bacillus Calmette–Guérin (BCG) administration on growth parameters in *ob/ob* mice.

	Group	
Parameters	Control	BCG
Food Intake (g/day)	4.44 ± 0.03	4.43 ± 0.07
Initial body weight (g)	29.9 ± 1.2	29.8 ± 1.0
Final body weight (g)	42.6 ± 0.8	41.4 ± 0.8
Liver weight (g/100g body weight)	6.90 ± 0.22	7.33 ± 0.11
Total WAT^†^ (g/100g body weight)	23.3 ± 1.1	22.8 ± 0.7
Epididymal WAT (g/100g body weight)	6.35 ± 0.34	5.38 ± 0.20*
Perirenal WAT (g/100g body weight)	3.63 ± 0.16	3.86 ± 0.21
Omental WAT (g/100g body weight)	3.25 ± 0.08	3.28 ± 0.08
Subcutaneous WAT (g/100g body weight)	10.1 ± 0.8	10.3 ± 0.4

WAT, white adipose tissue.

^†^Total white adipose tissue weight represents the sum of abdominal and subcutaneous white adipose tissue weights.

Each value is the mean ± SEM for six mice. Asterisk shows significant differences when compared to the control group by student *t-*test (*, *P* < 0.05).

**Table 2:** Effects of Bacillus Calmette–Guérin (BCG) administration on serum parameters in *ob/ob* mice.

	Group	
Parameters	Control	BCG
TG (mg/dL)	112 ± 31	61.1 ± 4.3
TC (mg/dL)	97.2 ± 7.0	84.3 ± 7.0
FFA (μEq/dL)	861 ± 69	789 ± 60
TNF-α (pg/mL)	n.d.	146 ± 26**
IFN-γ (pg/mL)	n.d.	58.9 ± 7.2**
MCP-1 (pg/mL)	92.4 ± 11.9	238 ± 19**
IL-6 (pg/mL)	1.20 ± 0.36	6.05 ± 1.28**
β-Hydroxybutyrate	2.44 ± 0.84	0.99 ± 0.36

TG, triglyceride; TC, total cholesterol; FFA, free fatty acids. TNF-α, tumor necrosis factor-α; IFN-γ, interferon-γ; MCP-1, monocyte chemoattractant protein-1; IL-6, interleukin-6.

Each value represents as the means ± SEM for six mice. n.d., not detectable. *Asterisk* shows significant differences when compared to the control group by student *t*-test (**, *P* < 0.01).

### Effect of BCG on the development of NAFLD

**Fig 1:**
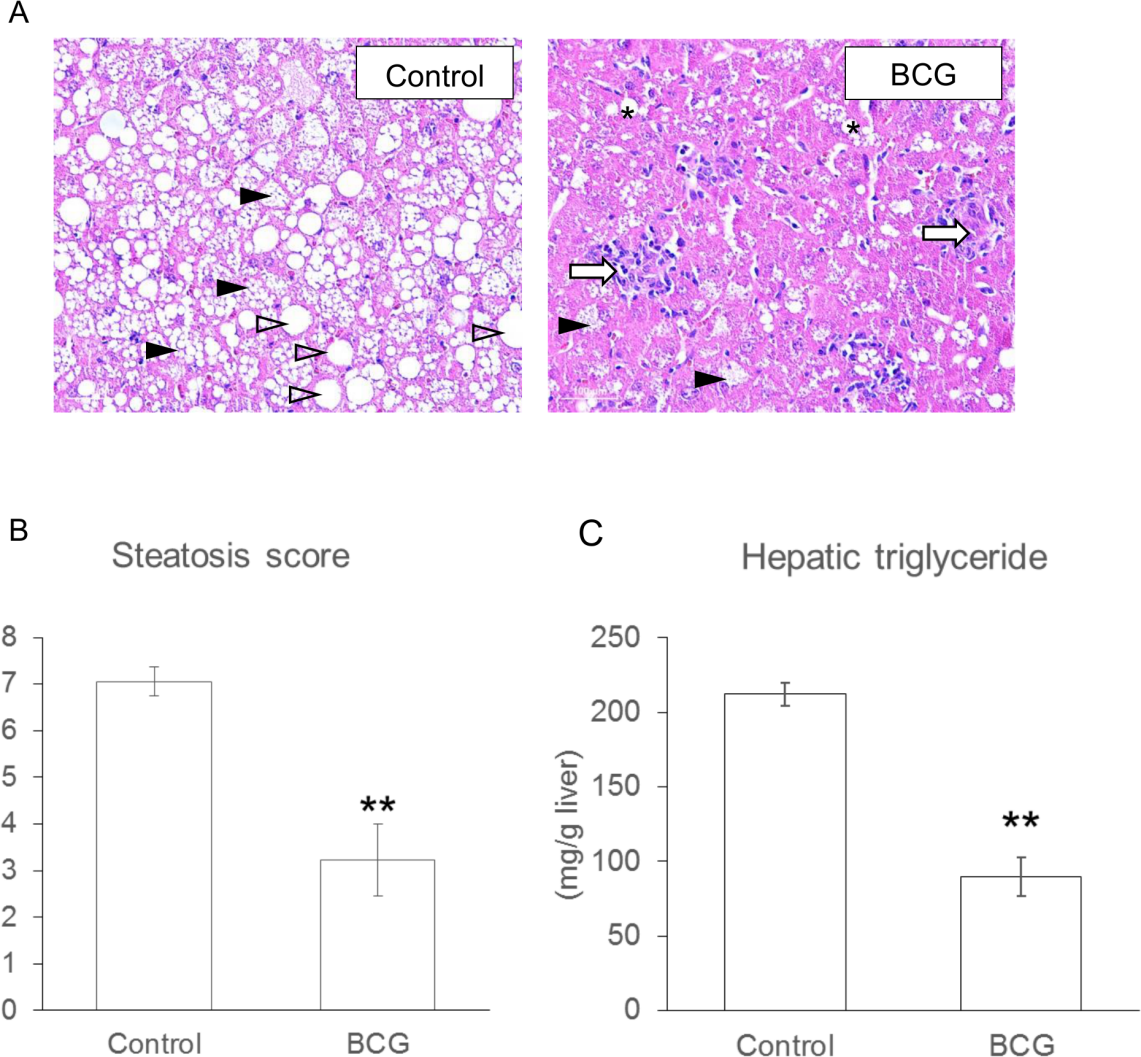
Effects of Bacillus Calmette–Guérin (BCG) on the development of NAFLD in *ob/ob* Mice. (A) Representative HE histology of the liver from control or BCG-treated *ob/ob* mice are demonstrated. Solid and open arrow heads indicate examples of macrovesicular and microvesicular steatosis, grrespectively. White arrows in the BCG group indicates granulomatous lesions induced by BCG. Steatosis score (B) and hepatic triglyceride content (C) is represented as the mean ± SEM for six mice. Asterisk shows the significant differences when compared to the control group (**, *P* < 0.01).

The macrovesicular and microvesicular lesions in the BCG-treated *ob/ob* mice tended to be smaller than those in the control *ob/ob* mice (Fig 1A). Steatosis score in the BCG group was significantly lower than that in the control group (Fig 1B). Although serum lipid levels and relative liver weights were comparable between the experimental groups (Tables 1 and 2), hepatic TG accumulation was significantly reduced in the BCG group compared with the control group (Fig 1C). A similar result was seen for hepatic TC levels (Control group, 5.22±0.32 mg/g liver; BCG group, 2.84±0.25 mg/g liver, *P* < 0.01).

### Effect of BCG on hepatic lipid metabolism

**Fig 2:**
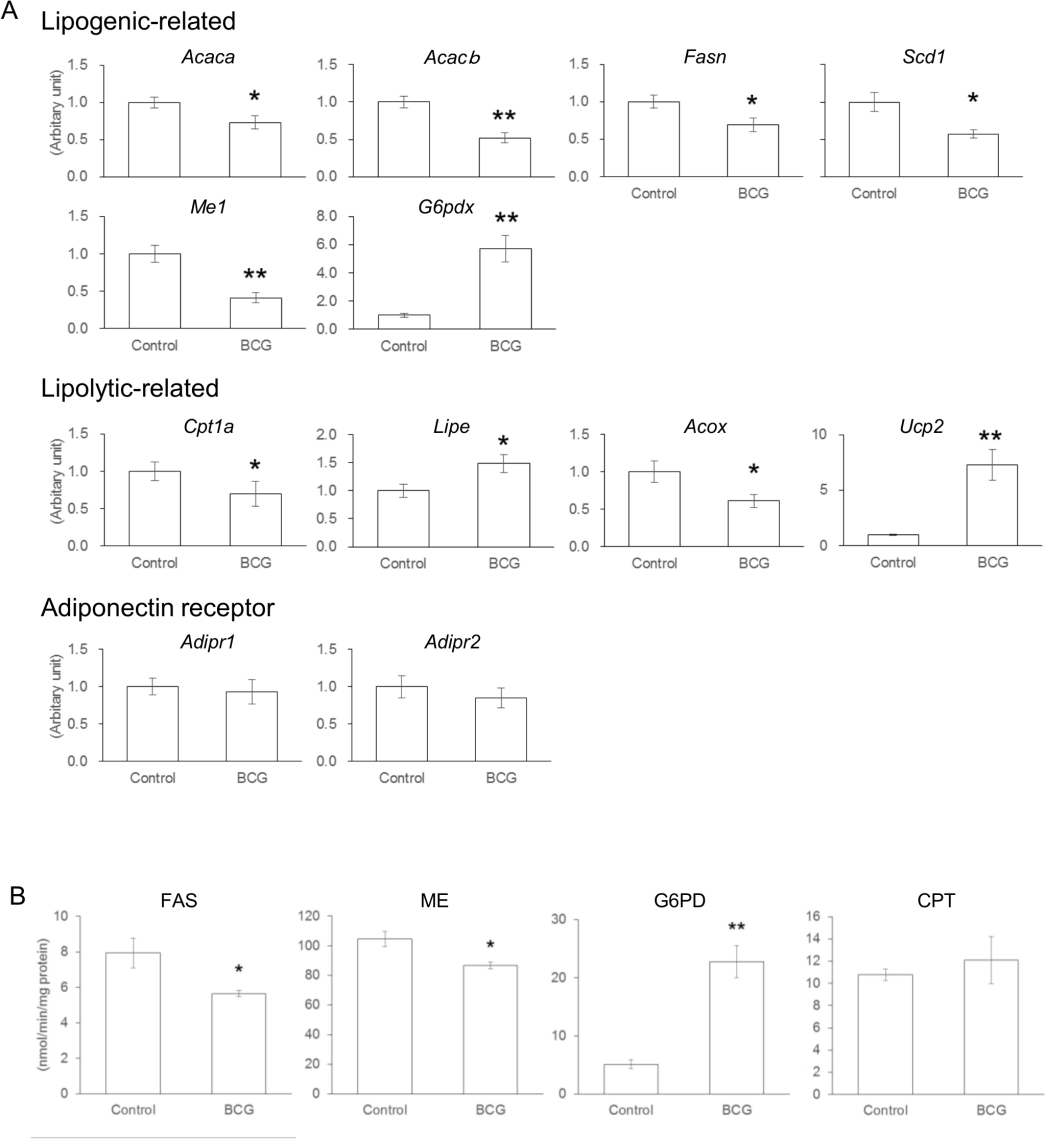
Effects of Bacillus Calmette–Guérin (BCG) on Lipid Metabolism in the Livers of *ob/ob* Mice. (A) mRNA levels of genes related to lipid metabolism in the liver. *Acaca*, acetyl-CoA carboxylase alpha; *Acacb*, acetyl-CoA carboxylase beta; *Fasn*, fatty acid synthase; *Scd*1, steroyl-CoA desaturase 1; *Me1*, malic enzyme 1; G6pdx, glucose-6-phosphate dehydrogenase; *Cpt1a*, carnitine palmitoyltransferase 1a; *Lipe*, hormone sensitive lipase; *Acox*, acyl-CoA oxidase 1; *Ucp2*, uncoupling protein 2; *Adipor*, adiponectin receptor. (B) The key lipogenic and lipolytic enzyme activities in the liver. Each value represents the mean ± SEM for six mice. Asterisk shows the significant differences when compared to the control group (*, *P* < 0.05; **, *P* < 0.01).

To understand the effect of BCG administration on hepatic lipid metabolism in *ob/ob* mice livers, we examined the mRNA levels of lipid metabolism-related genes and the activities of key lipogenic and lipolytic enzymes (Fig 2). Although the hepatic level of *G6pdx* mRNA was significantly increased, the mRNA levels of most lipogenic-related genes were significantly decreased in BCG-injected mice compared with control mice (Fig 2A). In particular, after BCG injection, there were significant decreases in *Cpt1a* and *Acox* mRNA; however, there were also significant increases in *Lipe* and *Ucp2* (Fig 2A). No significant differences were shown in adiponectin receptor gene expressions between the experimental groups (Fig 2A). The hepatic FAS and ME activities of BCG-treated mice were significantly lower compared with control mice (Fig 2B). Furthermore, G6PD activity was significantly increased in the BCG group compared with the control group. Thus, lipid metabolism-related gene expression was reflected in the enzymatic activity in the liver.

### Effect of BCG on serum adiponectin, insulin, and glucose levels

**Fig 3:**
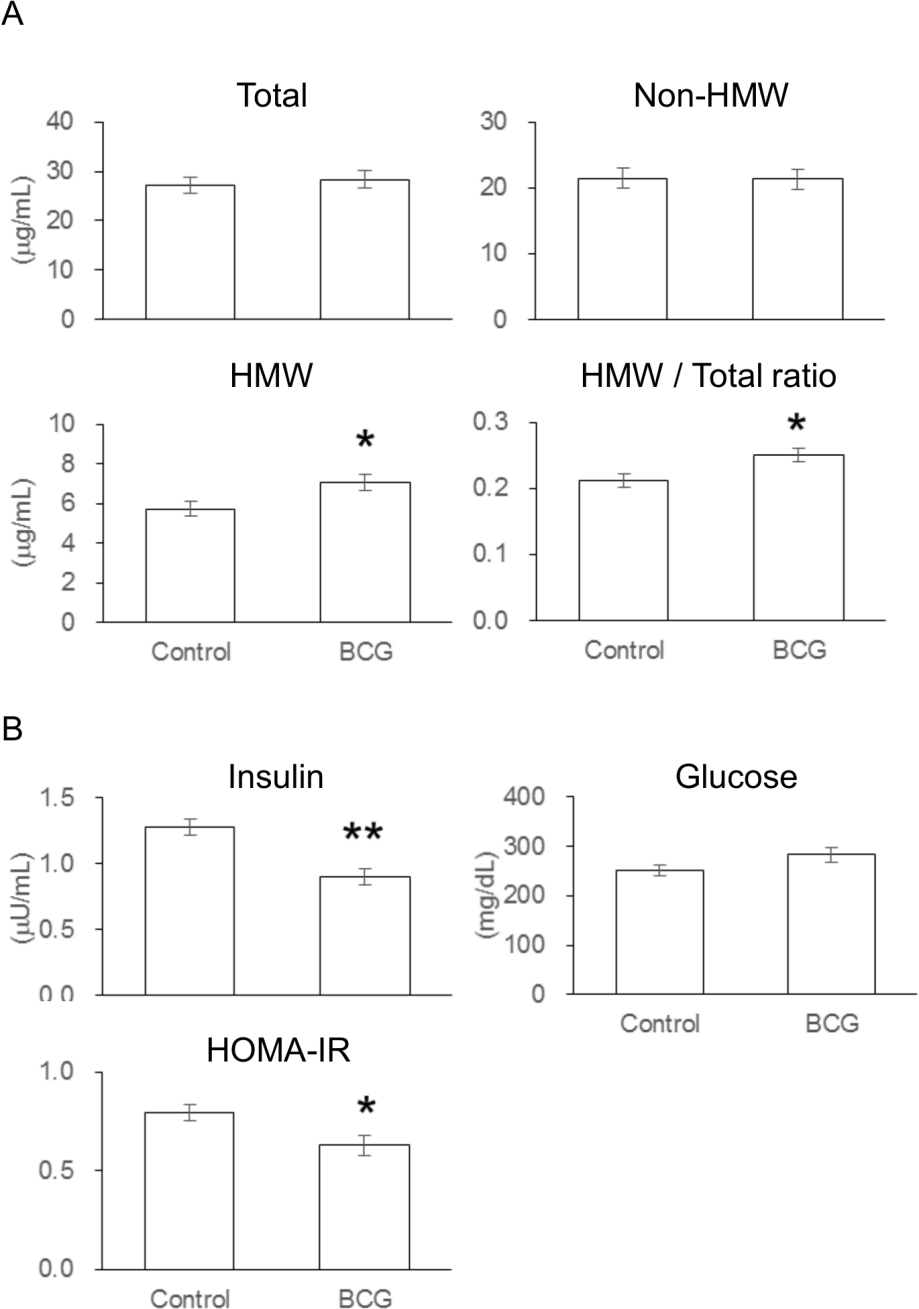
Effects of Bacillus Calmette–Guérin (BCG) on Serum Adiponectin Levels and Insulin Sensitivity in *ob/ob* Mice. (A) Serum levels of total, high molecular weight (HMW), and non-HMW adiponectin. The non-HMW adiponectin level was calculated as total adiponectin level–high HMW adiponectin. (B) Serum glucose and insulin levels. Homeostasis model assessment of insulin resistance (HOMA-IR) was calculated as follows: fasting glucose (mg/dL) × fasting insulin (μU/mL)/405. Each value represents the mean ± SEM for six mice. Asterisk shows significant differences when compared to the control group (*, *P* < 0.05; **, *P* < 0.01).

It is well known that IR is a major feature of NAFLD [30]. To understand the effect of BCG on IR, we examined the useful predictors of IR: homeostasis model assessment of insulin resistance (HOMA-IR) and the ratio of HMW to total adiponectin [31, 32]. Serum total adiponectin levels were largely comparable between experimental groups (Fig 3A). However, the levels of HMW adiponectin and the ratio of HMW to total adiponectin in the BCG group were significantly higher compared with the control group. No significant differences were observed in non-HMW adiponectin levels between the experimental groups. Administration of BCG reduced serum insulin levels, but did not affect serum glucose levels in *ob/ob* mice (Fig 3B). It was observed that HOMA-IR, which was calculated as {[fasting glucose (mg/dL) × fasting insulin (μU/mL)] / 405} was significantly decreased in the BCG group compared with the control group.

### Effect of BCG on lipogenesis and palmitate-induced stress and insulin tolerance in HepG2 cells

**Fig 4:**
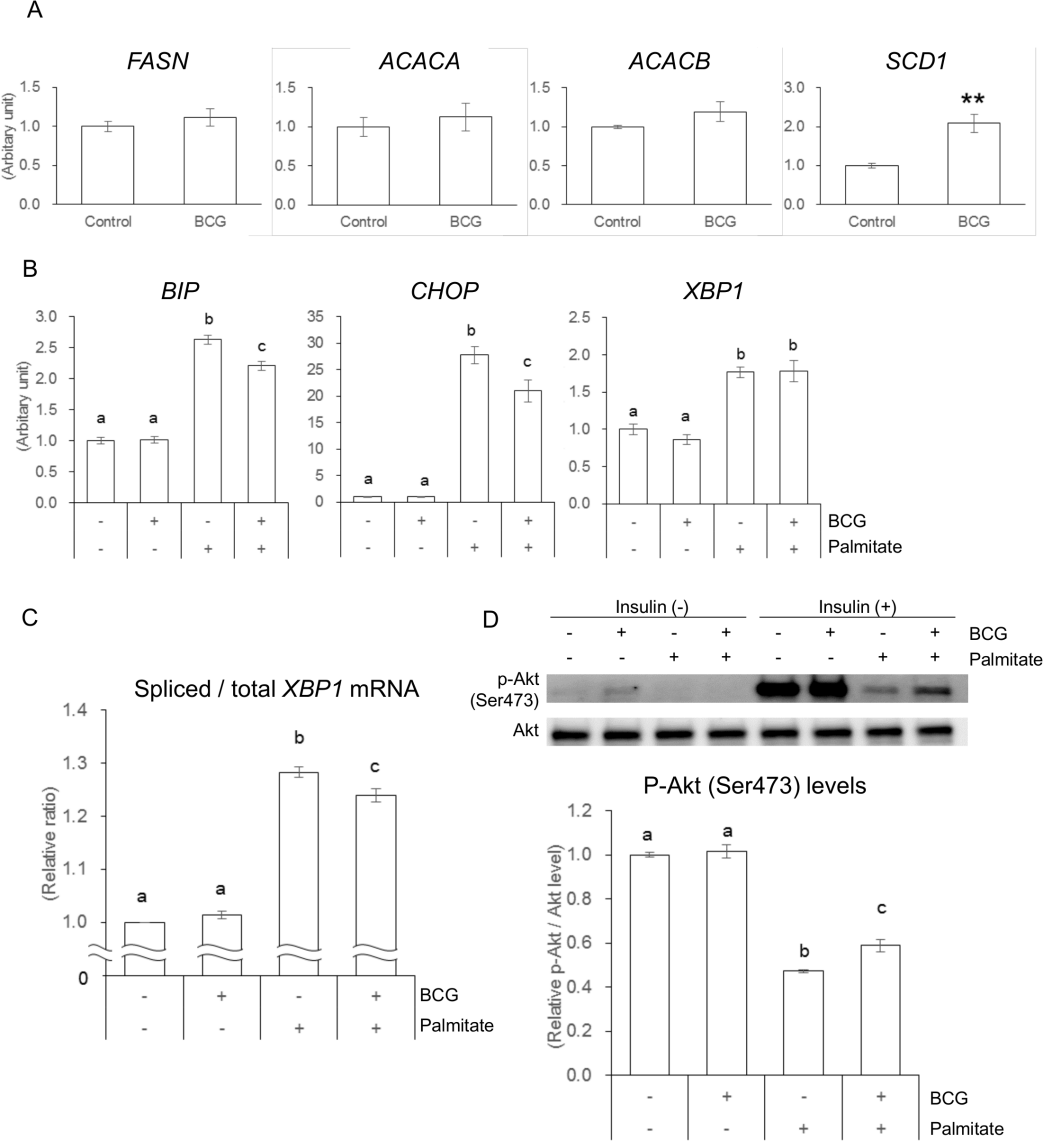
Effect of Bacillus Calmette–Guérin (BCG) on lipogenesis and Palmitate-induced Endplasmic Reticulum Stress and Insulin Tolerance in HepG2 Cells. (A)The mRNA levels of lipogenic-related genes. (B) The mRNA levels of ER stress-related genes. (C) Relative ratios of spliced/total *XBP1* mRNA. (D) The phosphorylation state of Akt (Ser473). Each value represents the mean ± SEM from at least three independent experiments. Different letters indicate significant differences among each experimental group (*P* < 0.05).

Based on the results in a murine model of *ob/ob* mice, we tested the direct effect of BCG on mRNA levels of lipogenic-related genes in HepG2 cells. Although a significant increase in *SCD1* mRNA level was shown, BCG treatment did not affect mRNA levels of *FASN*, *ACACA* and *ACACB* (Fig 4A). HepG2 cells exposed to palmitate showed an increase in the mRNA levels of endoplasmic reticulum (ER) stress-related genes (Fig 4B). Moreover, treatment with BCG alleviated the increases in *BIP* and *CHOP*, but not *XBP1*. Although no significant differences were observed in *XBP1* mRNA levels, BCG treatment alleviated the palmitate-induced increases in the ratio of spliced *XBP1* mRNA (Fig 4C), which is one of the indicators of ER stress [33]. To determine the insulin response in HepG2 cells exposed to palmitate, we next examined the insulin-induced Akt phosphorylation. The Akt phosphorylation was impaired by palmitate exposure (Fig 4D). The impaired serine phosphorylation of Akt was significantly ameliorated in HepG2 cells treated with BCG (Fig 4D). This amelioration appears to improve insulin sensitivity in HepG2 cells exposed to palmitate.

## Discussion

In this study, we investigated the effects of BCG on the development of metabolic syndrome in *ob/ob* mice. The intravenous administration of BCG significantly decreased the weight of perirenal WAT, TG accumulation, and lipogenic enzyme activity in the liver. The BCG administration also decreased serum insulin level, HOMA-IR, HMW adiponectin levels, and its ratio to total adiponectin in the serum, suggesting that the treatment ameliorated IR. Finally, in the presence of palmitate, BCG treatment significantly inhibited the expression of ER stress-related genesand alleviated the impaired insulin-stimulated Akt phosphorylation *in vitro*. These results suggest that BCG administration alleviated the NAFLD by inhibiting fatty acid-induced ER stress in livers of the *ob/ob* mice which resulted in amelioration of IR.

Abnormalities of hepatic lipid metabolism are largely attributable to hepatic fat accumulation, which represents the first hit in a “two-hit” hypothesis for the pathogenesis of NAFLD [34]. In the present study, both the mRNA levels and the enzymatic activities of most lipogenic-related genes were significantly decreased in the BCG group (Fig 2). In contrast, changes in the mRNA levels and the enzymatic activities of lipolytic-related genes did not follow the same metabolic consequences. The serum levels of β-hydroxybutyrate, a ketone body generated from fatty acid oxidation, were largely comparable between the experimental groups (Table 2). These data suggested that BCG injection at least partly inhibited the development of NAFLD by suppressing hepatic lipogenesis, although BCG did not directly affect the mRNA levels of lipogenenic-related genes in HepG2 cells (Fig 4A). Hepatic fat accumulation may be a primary event that leads to impaired insulin activity in the liver, and hepatic IR may be strongly linked to the development of NAFLD [35]. It is well known that the serum levels of inflammatory adipokines such as MCP-1, TNF-*α*, and IL-6 are increased in obese individuals, and could lead to impaired insulin sensitivity. MCP-1 plays an important role in macrophage infiltration into adipose tissues, which causes IR in adipocytes [36]. TNF-*α* and IL-6 have also been demonstrated to mediate IR partly through the suppression of insulin signal transduction [37, 38]. The important role of IFN-*γ* in regulating IR in obesity was suggested because insulin sensitivity in obese IFN-γ-knockout animals improved in comparison with obese wild-type control animals [39]. The production of inflammatory cytokines and chemokines is usually induced by viral and bacterial infections, and several studies have reported that metabolic syndrome was significantly associated with exposure to infectious agents [40–42]. BCG administration induces dramatic increases in serum inflammatory cytokine levels, leading to an expected aggravation of metabolic syndrome in *ob/ob* mice, which shares many features with NAFLD in humans [43]. However, contrary to our expectations, intravenous BCG alleviated the development of NAFLD in *ob/ob* mice (Fig 1).

Increases in plasma fatty acids led to hepatic IR [44]. Although serum FFA levels were largely comparable between the experimental groups in this study (Table 2), a significant decrease in HOMA-IR was shown in the BCG group (Fig 3B). Adiponectin, a highly abundant and insulin-sensitizing adipokine, reportedly enhances hepatic insulin action [45]. Furthermore, visceral fat accumulation is negatively correlated with systemic adiponectin levels, and strongly linked to IR and NAFLD. However, in this study we found no significant differences in relative omental (visceral) fat weight and serum levels of total and non-HMW adiponectin between the experimental groups (Table 1 and Fig 3A), although serum HMW adiponectin levels were significantly higher in the BCG group than in the control group (Fig 3A). The HMW oligomer forms of adiponectin have recently been shown to possess the greatest insulin-sensitizing activity [31], and the ratio of HMW to total adiponectin has been reported to be more useful for predicting IR and the metabolic syndrome than the total adiponectin level [31]. Therefore, the changes of HOMA-IR and HMW to total adiponectin ratio in this study suggest that improved insulin sensitivity contributed to the improved NAFLD in *ob/ob* mice injected with BCG. The adipocytes are reported to be important candidates of Mtb persistence in nonprofessional phagocytes in nearly a decade [46, 47]. Recent studies have reported that Mtb infection induces inflammatory cytokine production in cultured 3T3-L1 adipocytes [48] and murine primary adipocytes [49], with the latter indicating that adiponectin production was increased by treatment with a live Mtb, but not killed Mtb [49]. Furthermore, the intravenous injection of paraformaldehyde-killed BCG did not affect the development of metabolic disorders in *ob/ob* mice. Thus, infection with live BCG injection rather than interaction with mycobacterial molecules appeared to modify the physiological properties of adipocytes in *ob/ob* mice. Despite these promising results, further studies are needed to clarify the effects of BCG treatment on adipocytes.

Obesity is associated with an increase in FFA influx into the circulation, which leads to enhance FFA uptake into multiple tissues, including the liver [50]. FFA affects on various signal pathways that inhibit intracellular action of insulin; therefore, it is assumed to play a pathogenic role in both systemic and hepatic IR. Although several underlying mechanisms have been reported to explain how fatty acids impair insulin action, ER stress has emerged as an important contributor to both systemic and hepatic IR [51, 52]. In response to ER stress, the cells activated the unfolded protein response (UPR) to maintain ER function. ER chaperon BIP is known as the master regulator of UPR, and its expression is used as a marker of ER stress [53]. Spliced *XBP1* mRNA encodes an active transcriptional factor that upregulates ER chaperon genes [54]. CHOP plays a role in disruption of ER function and apoptosis induced by long-chain saturated fatty acids [55]. Palmitate, one of the most abundant FFAs in blood, induce ER stress in various cell types [56], and a recent study has established a link between ER stress and palmitate-induced IR in HepG2 cells and mouse primary hepatocytes [57]. In the latter study, palmitate led to the specific inhibition of insulin-stimulated Akt phosphorylation (Ser473), which is independent of tyrosine phosphorylation of insulin receptor substrate 1, and the inhibition was released by attenuating of ER stress with chemical chaperones. Our *in vitro* studies of HepG2 cells indicated that BCG treatment significantly decreased both the expression of ER stress-related genes (Fig 4B) and the ratio of spliced *XBP1* mRNA (Fig 4C), although BCG treatment did not affect gene expression for IFN-*β*, which is an important cytokine in host defense, particularly against intracellular pathogens. Furthermore, in HepG2 cells cultured with palmitate, the insulin-stimulated phosphorylation level (Ser473) of Akt was higher in the BCG-treated group than in the untreated group (Fig 4D) without changes in glucose and FFA levels in the culture medium. Notably, XBP1 has been shown to promote the transcription of lipogenic genes and to bind to promoter regions of the *Scd1* and *Acacb* genes in the liver [58]. The ratio of spliced *XBP1* mRNA in HepG2 exposed to palmitate significantly decreases after BCG treatment (Fig 4C). Our *in vivo* study showed that there was a significant increase in hepatic mRNA levels of lipogenic *Scd1* and *Acacb* genes in mice after BCG injection (Fig 2A). Although further studies are needed to understand the underlying mechanisms by which BCG treatment attenuated palmitate-induced ER stress in liver, our data suggest that BCG administration protected the liver from palmitate induced-ER stress, thereby inhibiting hepatic IR and lipogenesis.

In conclusion, the results of the present study indicate that BCG administration significantly inhibited the development of NAFLD by affecting the physiological state of both hepatocytes and adipocytes in the *ob/ob* mice. However, further studies are needed to understand the underlying mechanisms by which BCG administration alleviates NAFLD more completely. In addition, the mechanism of metabolic modulation in the liver and adipocytes by the BCG inoculation is an important target for drug development against the metabolic syndrome.

## Supporting Information

S1 Fig Effects of BCG on food intakes of *ob/ob* mice. (TIF)

S2 Fig Effects of Bacillus Calmette–Guérin (BCG) on Culture Condition of HepG2 Cells. (TIF)

S3 Fig Effect of BCG on mRNA level of *IFNB1* gene in HepG2 cells. (TIF)

S1 Table Oligonucleotide primer sequences for real time polymerase chain reaction and analysis of XBP1 splicing. (DOCX)

S2 Table Effects of live and paraformaldehyde-killed BCG on *ob/ob* mice (n = 3 mice / group). (DOCX)
