# Supplementary figures and images for: Intravenous Mycobacterium Bovis Bacillus Calmette-Guérin Ameliorates Nonalcoholic Fatty Liver Disease in Obese, Diabetic ob/ob Mice
Source: PLoS One. 2015 Jun 3;10(6):e0128676. doi: 10.1371/journal.pone.0128676 (PMC4454685; doi:10.1371/journal.pone.0128676)

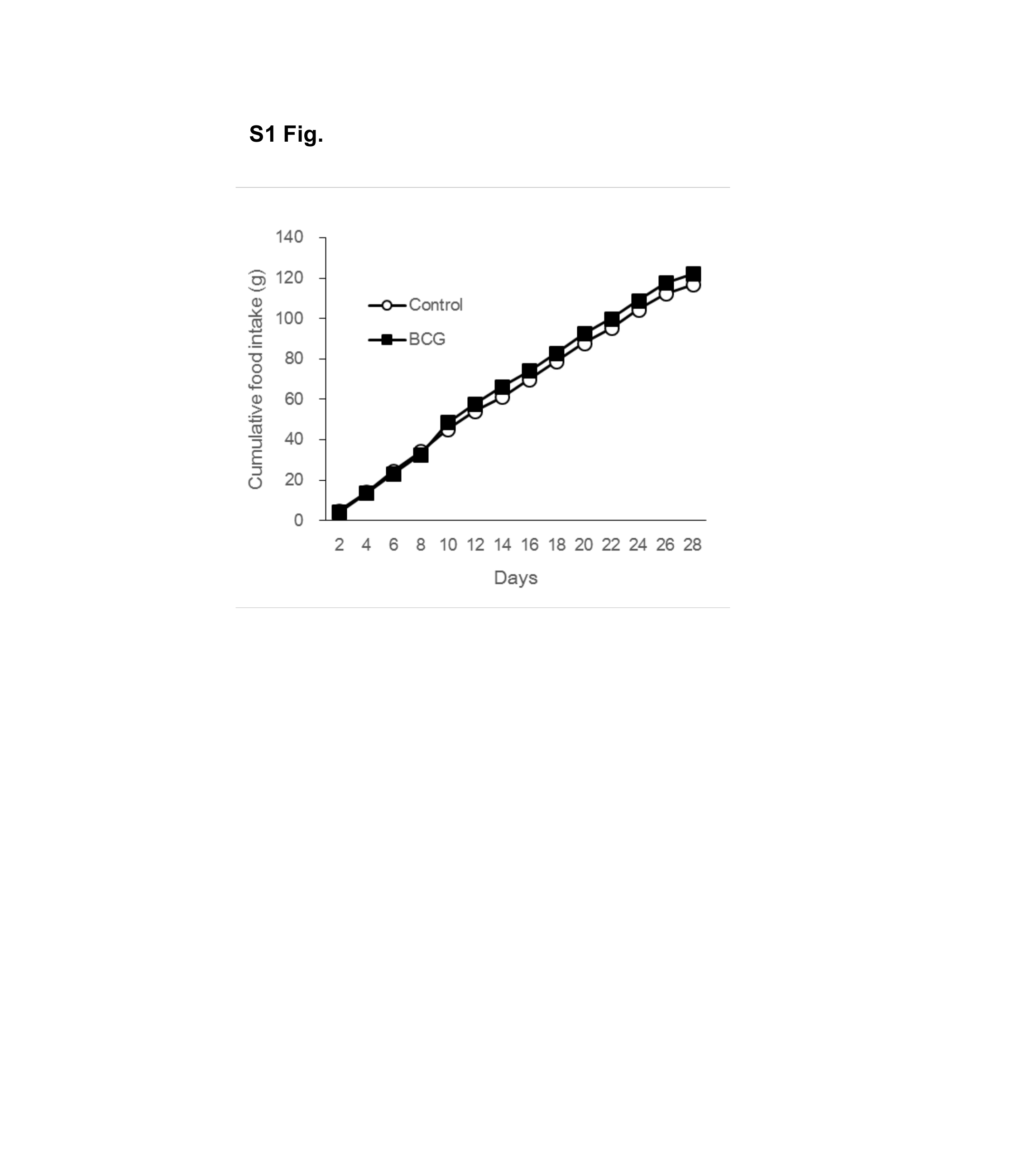

Supplement: S1 Fig — (TIF) [file pone.0128676.s001.tif]

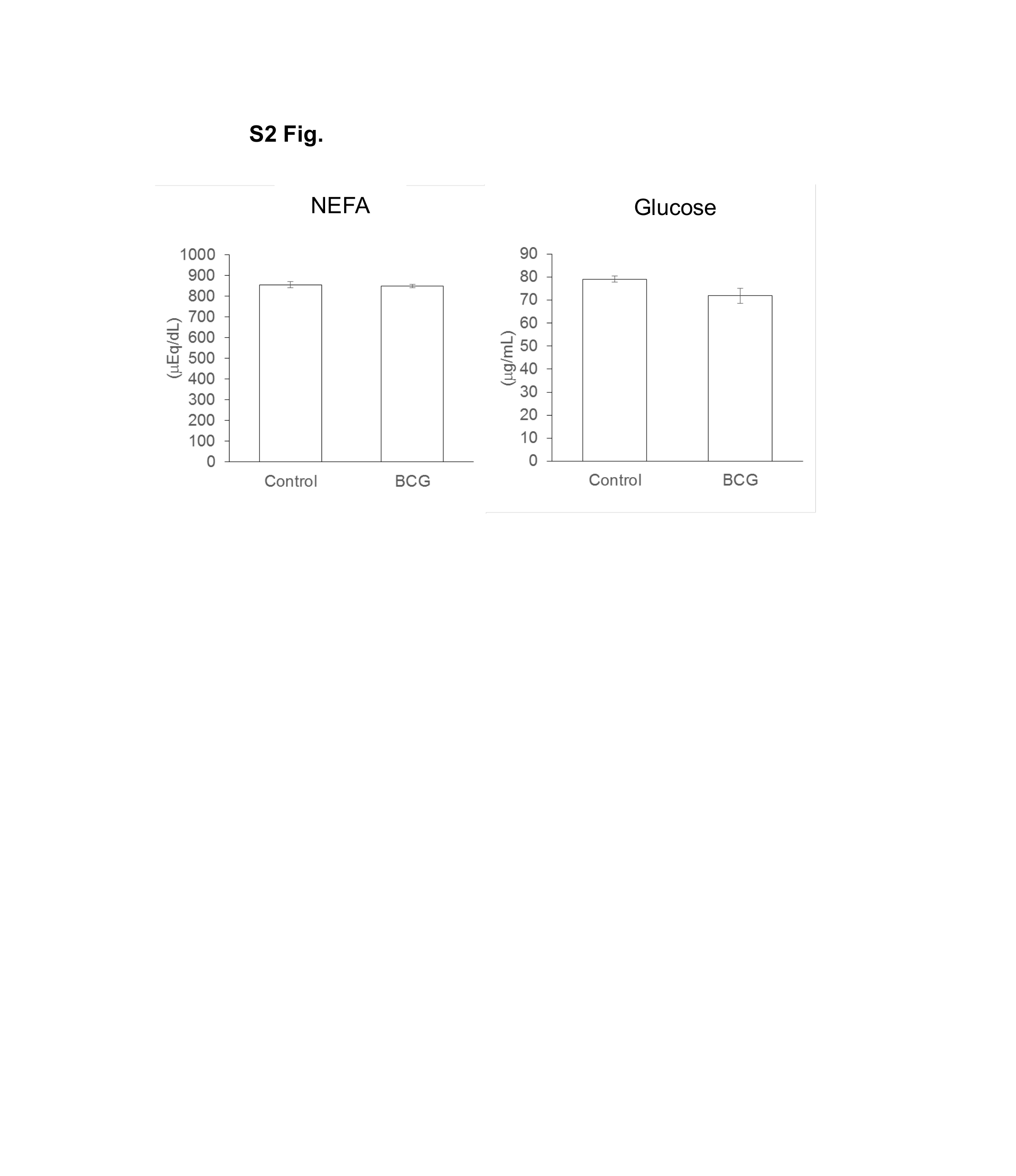

Supplement: S2 Fig — (TIF) [file pone.0128676.s002.tif]

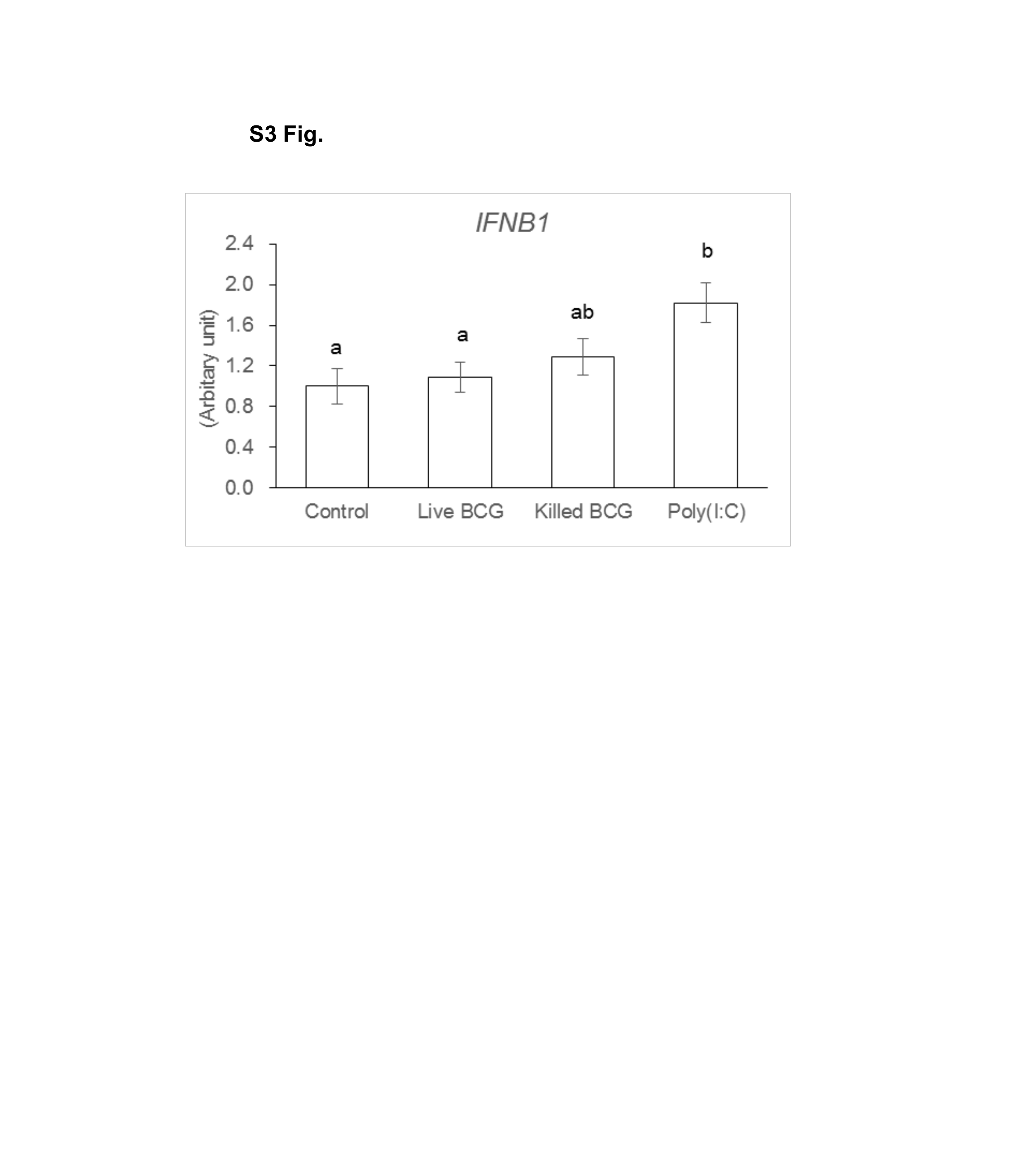

Supplement: S3 Fig — (TIF) [file pone.0128676.s003.tif]
