# Supplementary material for: Intravenous Mycobacterium Bovis Bacillus Calmette-Guérin Ameliorates Nonalcoholic Fatty Liver Disease in Obese, Diabetic ob/ob Mice
Source: PLoS One. 2015 Jun 3;10(6):e0128676. doi: 10.1371/journal.pone.0128676 (PMC4454685; doi:10.1371/journal.pone.0128676)
Supplement: S1 Table — (DOCX) [file pone.0128676.s004.docx]

**S1 Table**

| ***Target gene**** | **Species** | **Forward primer** | **Reverse primer** |
| --- | --- | --- | --- |
| *Rn18s* | Mouse | 5′- CGGACAGGATTGACAGATTG -3′ | 5′- CAAATCGCTCCACCAACTAA -3′ |
| *Acaca* | Mouse | 5′- GGCAGCTCTGGAGGTGTATG -3′ | 5′- TCCTTAAGCTGGCGGTGTT -3′ |
| *Acacb* | Mouse | 5′- CCCAGCCGAGTTTGTCACT -3′ | 5′- GGCGATGAGCACCTTCTCTA -3′ |
| *Fasn* | Mouse | 5′- TGCTCCCAGCTGCAGGC -3′ | 5′- GCCCGGTAGCTCTGGGTGTA -3′ |
| *Me1* | Mouse | 5′- GTCGTGCATCTCTCACAGAAG -3′ | 5′- TGAGGGCAGTTGGTTTTATCTTT -3′ |
| *G6pdx* | Mouse | 5′- GAACGCAAAGCTGAAGTGAGACT -3′ | 5′- TCATTACGCTTGCACTGTTGGT -3′ |
| *Scd1* | Mouse | 5′- CCGGAGACCCCTTAGATCGA -3′ | 5′- TAGCCTGTAAAAGATTTCTGCAAACC -3′ |
| *Cpt1a* | Mouse | 5′- AAAGATCAATCGGACCCTAGACA -3′ | 5′- CAGCGAGTAGCGCATAGTCA -3′ |
| Lipe | Mouse | 5′- GGTGACACTCGCAGAAGACAATA -3′ | 5′- GCCGCCGTGCTGTCTCT -3′ |
| *Acox* | Mouse | 5′- TCAACAGCCCAACTGTGACTTCCATTA -3′ | 5′- TCAGGTAGCCATTATCCATCTCTTCA -3′ |
| *Ucp2* | Mouse | 5′- CAGGTCACTGTGCCCTTACCAT -3′ | 5′- CACTACGTTCCAGGATCCCAAG -3′ |
| *Adipor1* | Mouse | 5′- TCTTCGGGATGTTCTTCCTGG -3′ | 5′- TTTGGAAAAAGTCCGAGAGACC -3′ |
| *Adipor2* | Mouse | 5′- CCTTTCGGGCCTGTTTTAAGA -3′ | 5′- GAGTGGCAGTACACCGTGTG -3′ |
| *ACTB* | Human | 5′- TCACCGAGCGCGGCT -3′ | 5′- TAATGTCACGCACGATTTCCC -3′ |
| *FASN* | Human | 5′- TCACCGAGCGCGGCT -3′ | 5′- TAATGTCACGCACGATTTCCC -3′ |
| *SCD1* | Human | 5′- TCACCGAGCGCGGCT -3′ | 5′- TAATGTCACGCACGATTTCCC -3′ |
| *ACACA* | Human | 5′- TCACCGAGCGCGGCT -3′ | 5′- TAATGTCACGCACGATTTCCC -3′ |
| *ACACB* | Human | 5′- AGAAGACAAGAAGCAGGCAAAC -3′ | 5′- GTAGACTCACGAGATGAGCCA -3′ |
| IFNB1 | Human | 5′- AAGGCCAAGGAGTACAGTC -3′ | 5′- AGTTTCGGAGGTAACCTG -3′ |
| *HSPA5 (BIP)* | Human | 5′- CGAGGAGGAGGACAAGAAGG -3′ | 5′- CACCTTGAACGGCAAGAACT -3′ |
| *DDIT3 (CHOP)* | Human | 5′- TGCCTTTCTCTTCGGACACT -3′ | 5′- TGTGACCTCTGCTGGTTCTG -3′ |
| *XBP1* | Human | 5′- AGTTGTCACCCCTCCAGAACATC -3′ | 5′- GGTCCAAGTTGTCCAGAATGCC -3′ |
| *XBP1* (for analysis of splising) | Human | 5′- AAACAGAGTAGCAGCTCAGACTGC -3′ | 5′- GTATCTCTAAGACTAGGGGCTTGGTA -3′ |
| *STAT1* |  | 5′- CTGCTGCGGTTCAGTGAGAG -3′ | 5′- GGTTCAACCGCATGGAAGTC -3′ |
