# Supplementary material for: Intravenous Mycobacterium Bovis Bacillus Calmette-Guérin Ameliorates Nonalcoholic Fatty Liver Disease in Obese, Diabetic ob/ob Mice
Source: PLoS One. 2015 Jun 3;10(6):e0128676. doi: 10.1371/journal.pone.0128676 (PMC4454685; doi:10.1371/journal.pone.0128676)
Supplement: S2 Table — (DOCX) [file pone.0128676.s005.docx]

**S2 Table**

|  | **Group** | | |
| --- | --- | --- | --- |
| **Parameters** | **Control** | **Live BCG** | **Killed BCG** |
| Hepatic TG (mg/g liver) | 212 ± 27 ^a^ | 94 ± 27 ^b^ | 260 ± 30 ^a^ |
| Serum total adiponectin (μg/mL) | 3.52 ± 0.21 | 4.16 ± 0.45 | 4.09 ± 0.27 |
| Serum HMW adiponectin (μg/mL) | 0.892 ± 0.107 ^a^ | 1.561 ± 0.253 ^b^ | 0.988 ± 0.280 ^ab^ |
| Serum HMW / total adiponectin ratio | 0.251 ± 0.019 ^a^ | 0.365 ± 0.028 ^b^ | 0.231 ± 0.047 ^a^ |
| Serum insulin (nU/mL) | 552 ± 10 ^a^ | 316 ± 51 ^b^ | 411 ± 50 ^ab^ |
| Serum glucose (mg/dL) | 366 ± 30 ^a^ | 339 ± 39 ^a^ | 470 ± 24 ^b^ |
| HOM-IR | 0.485 ± 0.065 ^a^ | 0.280 ± 0.069 ^b^ | 0.486 ± 0.077 ^ab^ |
